# Supplementary material for: Abnormalities of Hippocampal Subfield and Amygdalar Nuclei Volumes and Clinical Correlates in Behavioral Variant Frontotemporal Dementia with Obsessive–Compulsive Behavior—A Pilot Study
Source: Brain Sci. 2023 Nov 11;13(11):1582. doi: 10.3390/brainsci13111582 (PMC10669726; doi:10.3390/brainsci13111582)
Supplement: Supplementary file 1 [file brainsci-13-01582-s001.zip › brainsci-2655224-supplementary.pdf]

Supplementary Figure S1: Y-BOCS and left CA2/3 body volume mediation model; a, b, c, and c' are path coefficients representing unstandardized regression weights and standard errors (in parentheses). The c path coefficient represents the total effect of the left CA2/3 body volume on Y-BOCS score. The c' path coefficient refers to the direct effect of the left CA2/3 body volume score on Y-BOCS score. All analyzed a, b, and c paths with solid line were significant. CA: cornu ammonis; Y-BOCS:the Yale–Brown Obsessive–Compulsive Scale; OFC: orbitofrontal cortex.

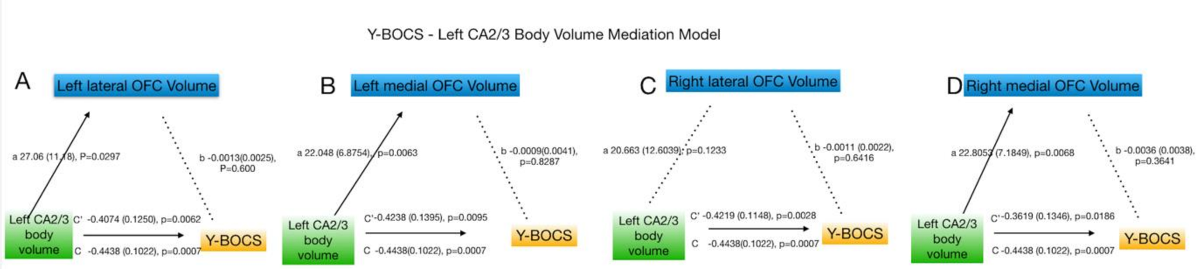

Supplementary Table S1. Normality tests (Kolmogorov-Smirnoff and Shapiro-Wilk test)

		Kolmogorov-Smirnoff	Shapiro-Wilk test
Age, y		0,096	0137
education		0.002	0.009
MMSE		<0.001	<0.001
TIV		.127	.169
Hippocampal subfield volume			
Left	Parasubiculum	.200	.852
	Presubiculum-head	.200	.546
	Presubiculum-body	.200	.586
	Subiculum-head	.200	.943
	Subiculum-body	.185	.684
	CA1-head	.200	.958
	CA1-body	.200	.961
	CA2/3-head	.200	.198
	CA2/3-body	.200	.910
	CA4-head	.143	.078
	CA4-body	.126	.043
	GC-ML-DG-head	.200	.461
	GC-ML-DG-body	.168	.228
	Molecular_layer_HP-head	.200	.880
	Molecular_layer_HP-body	.200	.356
	HATA	.200	.531
	Fimbria	.200	.648
	Hippocampal tail	.200	.785
	Hippocampal fissure	.110	.631
	Whole hippocampus	.116	.292
Right	Parasubiculum	.200	.130
	Presubiculum-head	.136	.599
	Presubiculum-body	.200	.643
	Subiculum-head	.200	.971
	Subiculum-body	.200	.245
	CA1-head	.200	.776
	CA1-body	.200	.481

CA2/3-head	.200	.951
CA2/3-body	.200	.631
CA4-head	.200	.669
CA4-body	.200	.584
GC-ML-DG-head	.200	.567
GC-ML-DG-body	.200	.348
Molecular_layer_HP-head	.200	.810
Molecular_layer_HP-body	.200	.296
HATA	.200	.933
Fimbria	.200	.183
Hippocampal tail	.200	.291
Hippocampal-fissure	.200	.623
Whole hippocampus	.200	.714

Supplementary Table S2. Simple mediation

Clinical severity	Hippocampal subfield volume	OFC volume	a	b	c	c’
Y-BOCS	Left CA2/3 body volume	Left lateral	27.06 (11.18), P=0.0297	-0.0013 (0.0025), P=0.6	-0.4438 (0.1022), p=0.0007	-0.4074 (0.1250), p=0.0062
		Left medial	22.048 (6.8754),, p=0.0063	-0.0009(0.0041), p=0.8287	-0.4438(0.1022), p=0.0007	-0.4238 (0.1395), p=0.0095
		Right lateral	20.663 (12.6039), p=0.1233	-0.0011 (0.0022),, p=0.6416	-0.4438(0.1022), p=0.0007	-0.4219 (0.1148), p=0.0028
		Right medial	22.8053 (7.1849),, p=0.0068	-0.0036 (0.0038),, p=0.3641	-0.4438 (0.1022), p=0.0007	-0.3619 (0.1346), p=0.0186
Total NPI score	Left GC-ML-DG body	Left lateral	25.9079 (10.2830), p=0.0245	-0.0017 (0.0021), p=0.4363	-0.4089 (0.0786), p=0.0001	-0.3658 (0.0960), p=0.0022
		Left medial	19.9578 (6.5265), p=0.0085	-0.0027 (0.0033), p=0.4297	-0.4089 (0.0786), p=0.0001	-0.3559 (0.1027), p=0.0042
		Right lateral	21.5522(11.4413), p=0.0805	-0.0012(0.0019), p=0.5304	-0.4089 (0.0786), p=0.0001	-0.3828 (0.0899), p=0.0009
		Right medial	19.7570 (7.0027), p=0.0136	-0.0041 (0.0029),, p=0.1839	-0.4089 (0.0786), p=0.0001	-0.3285 (0.0952), p=0.0043
	Left total hippocampal volumes	Left lateral	1.2033 (0.3841), p=0.0073	-0.0009 (0.0024), p=0.7211	-0.0162(0.0033), p=0.0002	-0.0152 (0.0044),, p=0.0044
		Left medial	0.9554(0.2254), p=0.0008	-0.0006 (0.0040), ,p=0.8918	-0.0162 (0.0033),	-0.0157 (0.0051),

					p=0.0002	p=0.0092
		Right lateral	0.9944 (0.4442), p=0.0420	-0.0007 (0.0020),, p=0.7643	-0.0162 (0.0033), p=0.0002	-0.0156 (0.0039) ,p=0.0017
		Right medial	0.9310 (0.2524), p=0.0024	-0.0030 (0.0035), p=0.4144	-0.0162 (0.0033), p=0.0002	-0.0135 (0.0047) ,p=0.0125
	Right total hippocampal volumes	Left lateral	1.4755 (0.5328), p=0.0151	-0.0033 (0.0028), p=0.2654	-0.0164 (0.0057), p=0.0120	-0.0116 (0.0070) ,p=0.1219
		Left medial	1.3331 (0.2760),, p=0.0003	-0.0056 (0.0053), P=0.3309	-0.0164 (0.0057), P=0.0120	-0.0090 (0.0093), P=0.3504
		Right lateral	1.3221 (0.5867), P=0.0408	-0.0020 (0.0026), P=0.4544	-0.0164 (0.0057), P=0.0120	-0.0138 (0.0068), P=0.0626
		Right medial	1.2402 (0.3317), P=0.0022	-0.0069 (0.0044), P=0.1352	-0.0164 (0.0057), P=0.0120	-0.0078 (0.0076), P=0.3249
FBI	Left molecular layer of the HP body	Left lateral	15.4311 (6.0821), P=0.0237	-0.0005 (0.0016), P=0.7557	-0.1602 (0.0342), P=0.0003	-0.1526 (0.0427), P=0.0034
		Left medial	12.2654 (3.7707), P=0.0058	-0.0003 (0.0025), P=0.8951	-0.1602 (0.0342), P=0.0003	-0.1560 (0.0469), P=0.0055
		Right lateral	11.0461 (6.9956), P=0.1367	-0.0007 (0.0013), P=0.6319	-0.1602 (0.0342), P=0.0003	-0.1529 (0.0381), P=0.0015

	Right medial	12.0008 (4.0915), P=0.0109	-0.0022 (0.0022), P=0.3486	-0.1602 (0.0342), P=0.0003	-0.1341 (0.0435), P=0.0087
Left CA4 body	Left lateral	31.1120 (10.5977), P=0.0108	-0.0003 (0.0018), P=0.8534	-0.2777 (0.0676), P=0.0011	-0.2674 (0.0890), P=0.0102
	Left medial	22.6522 (6.8724), P=0.0053	-0.0008 (0.0027), P=0.7700	-0.2777 (0.0676), P=0.0011	-0.22593 (0.0932), P=0.0155
	Right lateral	26.4179 (11.9948), P=0.0449	-0.0001 (0.0016), P=0.9692	-0.2777 (0.0676), P=0.0011	-0.2761 (0.0814), P=0.0048
	Right medial	23.5128 (7.1642), P=0.0055	-0.0023 (0.0025), P=0.3847	-0.2777 (0.0676), P=0.0011	-0.2240 (0.0905), P=0.0279
Left hippocampal tail	Left lateral	4.7654 (2.1852), P=0.0468	-0.0013 (0.0017), P=0.4411	-0.0491 (0.0135), P=0.0027	-0.0427 (0.0159), P=0.0184
	Left medial	3.8922 (1.3694), P=0.0130	-0.0019 (0.0027), P=0.4948	-0.0491 (0.0135), P=0.0027	-0.0417 (0.0173), P=0.0314
	Right lateral	3.5679 (2.4332), P=0.1647	-0.0011 (0.0015), P=0.4963	-0.0491 (0.0135), P=0.0027	-0.0435 (0.0148), P=0.0091
	Right medial	3.8619 (1.4616), P=0.0193	-0.0033 (0.0024), P=0.1934	-0.0491 (0.0135), P=0.0027	-0.0364 (0.0161), P=0.0413

The a, b, c, and c’ are path coefficients representing unstandardized regression weights and standard errors (in parentheses). The c path coefficient represents the total effect of the hippocampal subfield volume on clinical severity. The c’ path coefficient refers to the direct effect of the hippocampal subfield volume on clinical severity. All analyzed a, b, and c paths with solid line were significant. Y-BOCS, the Yale–Brown Obsessive–Compulsive Scale; NPI, Neuropsychiatric Inventory; FBI, Frontal Behavioral Inventory; CA, cornu ammonis; GC-ML-DG, granule cell molecular layer of dentate gyrus; HP, hippocampal; OFC: orbitofrontal cortex.
